# Commentary: Are There Indeed Spliced Peptides in the Immunopeptidome?

**DOI:** 10.1016/j.mcpro.2021.100158

**Published:** 2021-10-02

**Authors:** Michele Mishto

**Affiliations:** 1 Centre for Inflammation Biology and Cancer Immunology (CIBCI) & Peter Gorer Department of Immunobiology, King's College London, London, United Kingdom; 2 Francis Crick Institute, London, United Kingdom

**Keywords:** Proteasomes, peptide splicing, proteomics, in vitro digestions, mass spectrometry, T cell response, epitopes, HLA class I, APP, antigen processing and presentation, CTL, cytotoxic T lymphocyte, HLA-I & -II, human leukocyte antigen class I & II, PCPS, proteasome-catalyzed peptide splicing

## Abstract

Proteasome-generated spliced epitopes presented by HLA class I complexes are emerging targets for T cell targeted immunotherapies. Their identification by mass spectrometry triggered heated debates, which find a representative opinion in one of the two fronts in the recent perspective article by Arie Admon. Briefly, he suggests that proteasomes cannot efficiently catalyze such a reaction, and, thus, that all spliced peptides identified in HLA class I immunopeptidomes and other specimens are artifacts. This hypothesis is in contrast with *in vitro*, *in cellula*, and *in vivo* results published since the discovery of proteasome-catalyzed peptide splicing in 2004.

## Highlights

• Spliced peptides can be immunogenic.
• Spliced epitopes are opening up interesting opportunities for cancer immunotherapies.

## Graphical Abstract

**Figure:**
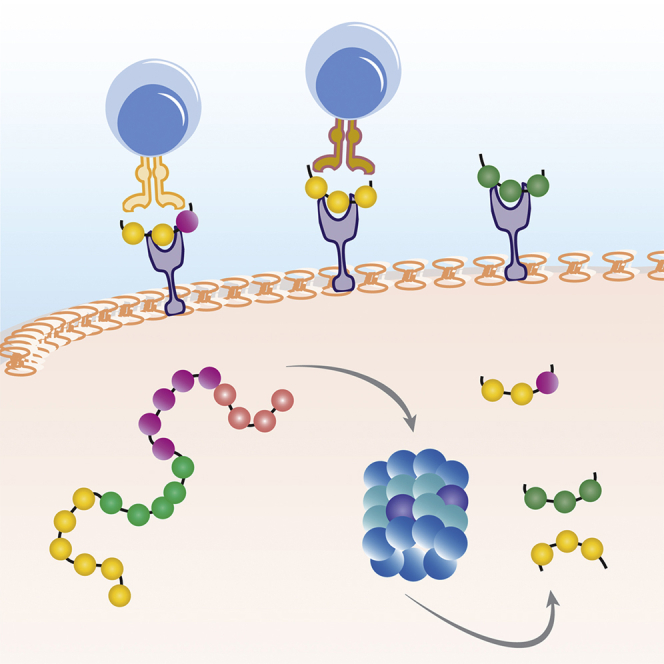

## In Brief

Poteasome-generated spliced peptides can be attractive targets for novel immunotherapies. Admon’s perspective “argued that peptide splicing is, at most, an extremely rare event and likely does not happen at all.” This statement is in contrast with data obtained through (i) MS on *in vitro* digestions of polypeptides and HLA class I immunopeptidomes, (ii) T cell clone assays with cell lines, (iii) *ex vivo* experiments in mouse models and human blood, (iv) anticancer immunotherapies in mouse models and human patients.

**Fig. 1:**
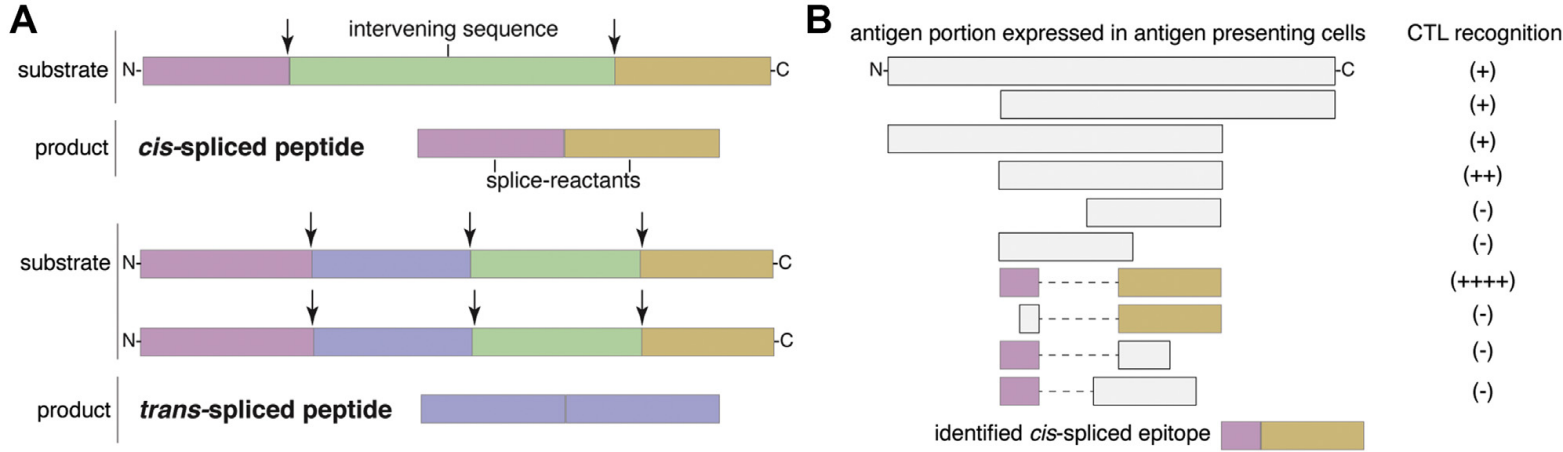
**Proteasome-generated spliced peptides and their original identification strategy.***A*, proteasomes can form spliced peptides through ligation of two noncontiguous splice reactants either derived from the same polypeptide molecule (*cis*-spliced peptides) or from two distinct molecules of the same protein or two distinct proteins (*trans*-spliced peptides). The two fragments, bound together by PCPS, are named splice reactants, and their junction is named splice site. The sequence segment between two splice reactants is called intervening sequence. *Arrows* represent the substrate cleavage sites used by proteasome catalytic Thr1. *B*, schematic of the original strategy that led to the identification of *cis*-spliced epitopes, *i.e.*, genetic truncation analysis using CTL clones as read-out system. Expression vectors containing different portions of an antigen are transfected into antigen presenting cells cocultured with the target CTL clone. The recognition of the expressed antigen is verified *via* cytokine secretion.

Through posttranslational peptide splicing, proteases can ligate noncontiguous sequences of a protein—thereby generating *cis*-spliced peptides—or distinct proteins, thus producing *trans*-spliced peptides (Fig. 1*A*). Posttranslational peptide splicing has been described within the context of Human Leukocyte Antigen class I & II (HLA-I & -II) antigen processing and presentation (APP) pathways (1). In the HLA-II APP pathways, cathepsin L has been suggested as one of the candidates catalyzing *trans*-peptide splicing (2), although other lysosomal proteases are known to be able to splice proteins (3, 4). In the HLA-I APP pathway, proteasomes have been described by many as generative of the most peptides—including posttranslationally *cis*-spliced peptides—bound to HLA-I complexes and presented to CD8^+^ T cells. *Cis*-spliced epitopes trigger a specific CD8^+^ T cell response against tumor-associated (5, 6, 7, 8, 9, 10, 11), Type1 Diabetes-associated (12), and *Listeria monocytogenes*-derived (13) antigens. They can also stimulate cross-recognition by cytotoxic T lymphocytes (CTLs) during infections (14, 15). A metastatic melanoma patient was successfully treated using an autologous tumor-infiltrating lymphocyte clone, which proved, in a later study, to be specific for a *cis*-spliced epitope rather than any nonspliced peptides derived from the melanoma-associated antigen (5, 16). Therapeutic successes targeting *cis*-spliced epitopes have also been described in nonobese diabetic/severe combined immunodeficient mice, wherein a CTL clone specific for an SP110-derived *cis*-spliced epitope could inhibit engraftment of human acute myelogenous leukemia cells (9, 17), as well as in peptide vaccination of a mouse model of glioblastoma (18). In the latter study, Fidanza and colleagues (18) suggested that most of the vaccine-derived epitopes that drove the therapeutic efficacy of that vaccine were spliced peptides produced by proteasomes. Although the theoretically very large sequence variability of proteasome-generated *cis*-spliced peptides can be a feature exploited by the immune system in the fight against cancer and infections, it has been preliminarily estimated to have a limited role in T cell tolerance and viral-driven autoimmunity (19, 20).

Despite a plethora of *in cellula*, *ex vivo*, and *in vivo* evidence supporting a sizeable presentation of spliced peptides to CTLs, and an involvement of these unconventional peptides in the immune response, researchers in this field are arguing about the frequency of spliced peptides in HLA-I immunopeptidomes (21, 22, 23). Indeed, after the initial studies on *cis*- and *trans*-spliced peptides in HLA-I immunopeptidomes (24, 25, 26), a barrage of contradicting studies has been published, leaving the community doubtful of the real relevance of spliced peptides in the immune response. A summary of these studies is reported in Admon’s perspective (23) published in this special issue, as well as other recent reviews (27). Admon (23), however, extends the narrative and “argued that peptide splicing is, at most, an extremely rare event and likely does not happen at all.” He based this claim on two main arguments. The first is biochemical and labels transpeptidation as a highly unlikely event because of the competition between water molecules and C-terminal splice reactants (Fig. 1*A*) for the nucleophilic attack to the acyl-enzyme intermediate. Transpeptidation is the biochemical process that recapitulates most of proteasome-catalyzed peptide splicing (PCPS) events, according to what was proposed by Vigneron and colleagues (8) and confirmed in other studies (9, 28, 29, 30). If Admon’s hypothesis was correct, only very few spliced peptides could be produced by proteasomes, and they would be generated in such small quantities that it is difficult to envisage any detection by common CTL assays. Consequently, the plethora of *in cellula*, *ex vivo*, and *in vivo* evidence briefly summarized in my *incipit* would either be technical artifacts, or the CTL response should have been addressed against nonspliced peptides (or unknown unconventional peptides) rather than the allegedly identified spliced epitopes. A CTL-mediated cross-recognition of spliced and nonspliced peptides has been described *ex vivo* (14, 15), and, in theory, might be the driver of the CTL response detected in peripheral blood of melanoma patients (6, 11). In contrast, the first tumor- and *L. monocytogenes*-associated *cis*-spliced epitopes reported in literature were identified with methods that excluded that the studied CTLs recognized both the putative *cis*-spliced epitopes and other nonspliced peptides derived from the same antigen (8, 9, 10, 13). In particular, in 2004, Yang, Yewdell, Van den Eynde, and coworkers (8, 10) had to construct an original hypothesis and suggest PCPS for the first time, because no other explanation was possible. In their experimental studies, they progressively reduced the length of antigens contained in expression vectors in antigen presenting cells cocultured with CTL clones, which were their read-out system. They arrived at a minimal antigen size, which excluded that canonical nonspliced peptides were recognized by the CTL clones. Then, only PCPS could explain the phenomenon, and thus they identified the minimal *cis*-spliced epitopes (Fig. 1*B*). In those pioneering studies (8, 9), the *in cellula* results were confirmed by biochemical experiments with purified proteasomes measured by mass spectrometry and other means, thereby revealing information on the biochemistry of PCPS.

The second argument of Admon’s hypothesis is based on bioinformatics considerations (23). The theoretically extremely large size of the *cis*- and *trans*-spliced peptide database represents a hurdle in their identification by mass spectrometry in complex samples such as HLA-I immunopeptidomes. The false discovery rate computation is likely underestimated by many of the current methods. None of the proposed methods performed an accurate evaluation of the method performance, such as precision and recall. Similar issues also characterize the identification of other unconventional peptides, such as peptides with a broad range of posttranslational modifications, those with single amino acid exchange, and peptides derived from putative noncoding regions. As mentioned by Admon in his perspective (23), a solution could be the comparison of all identified peptides of HLA-I immunopeptidomes with heavy stable isotope-label synthetic peptides. Although ideal, this solution is financially impractical, and, indeed, it has only been applied on target unconventional peptides (or small pools of unconventional peptides) in HLA-I immunopeptidomes (31). An alternative strategy could be studying PCPS in a simpler system, such as *in vitro* digestions of synthetic polypeptide substrates by purified proteasomes, measured by mass spectrometry. The theoretical number of *cis*- and *trans*-spliced peptides derived from synthetic polypeptides, which usually have a length between 20 and 40 amino acids for technical reasons, is relatively small; therefore, the statistical issues present in the analysis of HLA-I immunopeptidomes can be minimized in these kinds of assays. For a decade, there have been algorithms for the computation of theoretical *cis*- and *trans*-spliced peptides derived from synthetic polypeptides as well as methods for their identification and quantification through mass spectrometry (30, 32). The downside of this strategy is that the downstream steps of HLA-I APP pathway—*e.g.*, the aminopeptidase-mediated trimming of peptides produced by proteasomes—are not included, and thus the pool of peptide products represented only a subset of what could be presented by HLA-I complexes. Nonetheless, correspondence between this kind of *in vitro* experiment and *in cellula* and *in vivo* experiments has been established in various studies investigating HLA-I-restricted nonspliced and spliced epitopes (5, 6, 7, 8, 9, 13, 15, 28, 33, 34, 35, 36, 37, 38).

The largest database of nonspliced and spliced peptides produced by proteasomes in these *in vitro* assays has been published by Specht and colleagues (39) and contains almost 15,000 unique peptides. In this database, spliced peptides represent two-thirds of the peptide product variety (39). The proportion of spliced peptides in these samples is likely significantly lower than nonspliced peptides, as shown in quantitative studies (30, 40). In term of potential pitfalls, in this kind of *in vitro* digestions, the identification of spliced peptides could be misled by the presence of contaminations in the samples, especially peptide synthesis errors (31, 41, 42). However, Specht and colleagues (39) considered this issue and excluded, from the final peptide product list, any spliced peptide that was present as such, or as N-/C-terminal precursor in the negative control (t = 0 h). This should exclude that peptides generated by peptide hydrolysis of peptide synthesis errors inflated the pool of identified spliced peptide products (39). Furthermore, the experimental condition of this kind of assay might promote *cis*-PCPS, though experimental evidence supporting this hypothesis is not apparent. There is preliminary evidence, though, that these kinds of assays favor *trans*-PCPS between molecules of the same antigen (29, 30). Therefore, Specht and colleagues’ study (39), supported by other studies on smaller substrate datasets (15, 18, 42, 43, 44), contradicts the hypothesis of Admon (23) that peptide splicing is not catalyzed by proteasomes. Consequently, if PCPS is so frequent in these kinds of *in vitro* digestions, which have a controlled risk of peptide sequence misassignment, why should spliced peptides not be a sizeable fraction of HLA-I immunopeptidomes, as suggested by Admon (23)? One explanation may be that proteasomes are only marginally responsible for the production of HLA-I immunopeptidomes, as suggested by Admon and colleagues (45), although this hypothesis would clash with the common theory of HLA-I APP pathway (46, 47). Another explanation could be that spliced peptides are side products of proteasome activity, generated in such a low quantity that they cannot survive all filtering steps of HLA-I APP. This may be correct for many spliced peptides and explain the discrepancy in terms of spliced peptide frequency preliminarily detectable by comparing *in vitro* digestion assays and HLA-I immunopeptidomes, both measured *via* mass spectrometry. Nonetheless, through mass-spectrometry-independent strategies we have shown that *cis*-spliced and nonspliced epitopes can be presented in comparable amounts at the cell surface (6).

In conclusion, the growing research field of posttranslational peptide splicing is likely still in its infancy, and hypotheses raised in Admon’s perspective (23) have the merit of defining the extremes of the future debate. However, to paraphrase Admon’s comment (23), extraordinary claims—in this case, “peptide splicing is, at most, an extremely rare event and likely does not happen at all”—require extraordinary evidence.

## Conflict of interest

The author has no competing interests to declare.
